# 

**Published:** 1883-11

**Authors:** 


### CONTENTS:

ART. I.—Alimentation Among Savage and Civilized Peoples.....289

ART. II.—Pericementitis—Its Manifestations and Effects Upon the General System. By G. A. Mills, D. D. S.....312

ART. III.—Education of Mothers. By Mrs. M. W. J.....319

ART. IV.—Some Interesting Points in Oral Surgery.....321

ART. V.—The Necessity of Uniform Legislation.....326

ART. VI.—Ozone as an Anæsthetic and Hypnotic.....328

### EDITORIAL, ETC.

The Maryland State Dental Association.....330
University of Maryland Dental Department.....330
Aluminum Alloy.....331
Food Makes the Man.....331
United States Pharmacopœia.....332

### MONTHLY SUMMARY.

Smoothness of Dental Plates.....332
A Singular Case.....333
Bone Degeneration in the Insane.....334
How Do Parasites Produce Disease.....335
Transplation of Muscle in Man.....336

